# Supplementary material for: Identification of photoperiod-induced specific miRNAs in the adrenal glands of Sunite sheep (Ovis aries)
Source: Front Vet Sci. 2022 Jul 22;9:888207. doi: 10.3389/fvets.2022.888207 (PMC9354845; doi:10.3389/fvets.2022.888207)
Supplement: Supplementary file 1 [file Data_Sheet_1.ZIP › Supplementary file/miRDeep2 file.zip!/miRDeep2 file/Novel_Structure/LP42A1/Novel_481.pdf]

Provisional ID	:	NC_019460.2_7127
Score total	:	2.6
Score for star read(s)	:	-1.3
Score for read counts	:	0
Score for mfe	:	2.3
Score for randfold	:	1.6
Score for cons. seed	:	
Total read count	:	56
Mature read count	:	47
Loop read count	:	0
Star read count	:	9

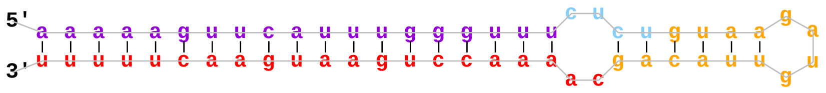

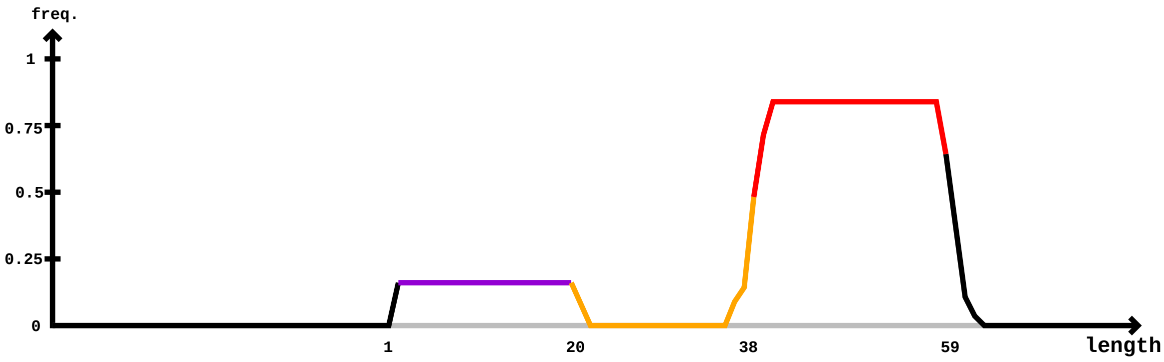

Star Mature

```
5'- cacugaggggagucacauaguauuggacuggccaaaaaaguucauuuggguuucucuguaagauguuacagcaaaaccugaaugaacuuuuuuggccaacccaauauuugu   -3'   obs
    cacugaggggagucacauaguauuggacuggccaaaaaaguucauuuggguuucucuguaagauguuacagcaaaaccugaaugaacuuuuuuggccaacccaauauuugu         exp
    .(((......))).(((.((((((((..(((((((((((((((((((((((((..((((((....))))))..)))))))))))))))))))))))))..)))))))))))   reads   mm   sample
    ..................................aaaaaguucGuuuggguuu..........................................................   6       1    seq
    ..................................aaaaaguucGuuuggguuuc.........................................................   3       1    seq
    .....................................................................agAaaaaccugaaugaacuuuu....................   5       1    seq
    ......................................................................gcaaaaccugaaugaacuuuu....................   1       0    seq
    ......................................................................gcaaaaccugaaugaacuuuuu...................   1       0    seq
    ......................................................................gGaaaaccugaaugaacuuuuu...................   1       1    seq
    .......................................................................Aaaaaccugaaugaacuuuu....................   4       1    seq
    .......................................................................Aaaaaccugaaugaacuuuuu...................   15      1    seq
    ........................................................................aaaacUugaaugaacuuuu....................   1       1    seq
    ........................................................................aaaaccugaaugaacuuuuuA..................   2       1    seq
    ........................................................................aaaaccAgaaugaacuuuuuu..................   2       1    seq
    ........................................................................aaaaUcugaaugaacuuuuuu..................   6       1    seq
    ........................................................................aaaaccugaaugaacuuuuuGgg................   2       1    seq
    .........................................................................aaaccugaaugaacuuuuu...................   1       0    seq
    .........................................................................aaaccCgaaugaacuuuuuu..................   2       1    seq
    .........................................................................aaaccCgaaugaacuuuuuug.................   4       1    seq
```
